# Sarcopenia, healthy living, and mortality in patients with chronic liver diseases

**DOI:** 10.1002/hep4.2061

**Published:** 2022-08-10

**Authors:** Catherine Van Dongen, James M. Paik, Michael Harring, Youssef Younossi, Jillian K. Price, Khaled Kabbara, Pegah Golabi, Zobair M. Younossi

**Affiliations:** ^1^ Betty and Guy Beatty Center for Integrated Research, Inova Health System Falls Church Virginia USA; ^2^ Center for Liver Disease, Department of Medicine Inova Fairfax Medical Campus Falls Church Virginia USA; ^3^ Center for Outcomes Research in Liver Diseases Washington District of Columbia USA; ^4^ Inova Medicine, Inova Health System Falls Church Virginia USA

## Abstract

Chronic liver diseases (CLDs) are associated with increased morbidity and mortality. Sarcopenia is an important complication of CLD that can be impacted by several modifiable risk factors. Our aim was to assess the associations between healthy living, sarcopenia, and long‐term outcomes among patients with CLD. We used the Third National Health and Nutrition Examination Survey data with National Death Index–linked mortality files. We used the American Heart Association's Life's Simple 7 (LS7) metrics as surrogates of healthy living. The study included 12,032 subjects (34.9% CLDs [0.5% hepatitis B virus (HBV), 1.8% hepatitis C virus (HCV), 5.7% alcohol‐associated liver disease (ALD), 26.9% nonalcoholic fatty liver disease (NAFLD)] and 65.1% controls). Prevalence of sarcopenia was higher among NAFLD than other CLDs and the controls (40.7% in NAFLD, 27.2% in ALD, 22.4% in HCV, 16.8% in HBV, and 18.5% in controls; *p* < 0.001). Among NAFLD and ALD, patients with sarcopenia were less likely to meet ideal LS7 metrics than those without sarcopenia. During 27 years of follow‐up, among 4 patients with CLDs and the controls, all‐cause cumulative mortality was highest among patients with HCV (35.2%), followed by ALD (34.7%) and NAFLD (29.6%). The presence of sarcopenia was associated with higher risk of all‐cause mortality only among subjects with NAFLD (hazard ratio [HR] 1.24; 95% confidence interval [CI] 1.01–1.54; *p* = 0.04). Among subjects with NAFLD, presence of sarcopenia was associated with higher risk of cardiovascular‐specific (HR 2.28 [1.71–3.05; *p* < 0.01]), cancer‐specific (HR 1.90 [1.37–2.65]; *p* < 0.01), diabetes‐specific (HR 6.42 [2.87–14.36]; *p* < 0.01), and liver‐specific mortality (HR 2.49 [1.08–5.76]; *p* = 0.04). The multivariable model showed that component of LS7 metrics that provided the strongest protection against sarcopenia were ideal body mass index, ideal blood pressure, ideal physical activity, and ideal glycemic control among subjects with NAFLD subjects. *Conclusions*: Among subjects with NAFLD, sarcopenia is associated with a higher risk of all‐cause mortality and liver mortality. Attainment of ideal LS7 metrics provides protection against sarcopenia in NAFLD.

## Graphical abstract

Chronic liver diseases (CLDs) are associated with increased morbidity and mortality. Sarcopenia is an important complication of CLD. Among CLDs, NAFLD and ALD patients have worse LS7 patterns. Among NAFLD subjects, sarcopenia is associated with a higher risk of all‐cause mortality (especially among lean NAFLD) and liver mortality (especially among females). Attainment of ideal LS7 metrics provides protection against sarcopenia in NAFLD.

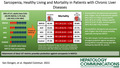

## INTRODUCTION

Globally, chronic liver disease (CLD) is a major cause of morbidity and mortality accounting for 3.5% of all deaths.^[^ ^1^ ^]^ It is estimated that about 1.8% of the adult US population (2018) is affected by CLD, and the prevalence of CLD is rising in the United States and globally.^[^ ^2^, ^3^, ^4^, ^5^ ^]^

Although viral hepatitis B (HBV) and C (HCV) have been recognized as important causes of liver disease, alcohol‐associated liver disease (ALD) and nonalcoholic fatty liver disease (NAFLD) are major contributors to the global burden of CLD.^[^ ^3^ ^]^ An important complication of advanced liver disease is sarcopenia, which is characterized by progressive and generalized loss of skeletal muscle mass, strength, and function.^[^ ^6^, ^7^ ^]^ The current evidence suggests that sarcopenia could potentially worsen the prognosis of patients with CLD.^[^ ^8^ ^]^

The underlying cause of sarcopenia among patients with advanced CLD is multifactorial. In addition to the catabolic state associated with cirrhosis, other factors may be contributing to the development and severity of sarcopenia. Some of these factors could be captured by the American Heart Association's (AHA) proposed Life's Simple 7 (LS7) health metrics. LS7 is a set of health factors that are surrogates of healthy living and can inform clinicians about the risk of developing CVD.^[^ ^9^ ^]^ Of the seven health metrics, four are ideal health behaviors (not smoking, body mass index [BMI] maintenance, physical activity level, and diet) and three are ideal health factors (blood pressure [BP], blood cholesterol, and blood glucose).^[^ ^9^ ^]^ In this context, maintenance of all seven metrics has been shown to improve long‐term health outcomes, especially all‐cause mortality, cancer mortality, and CVD mortality rates among the general US population.^[^ ^9^ ^]^ In the current study, our aim was to assess the impact of LS7 health metrics on patients with different types of CLD with or without sarcopenia.

## METHODS

### Study population

Study data were obtained from health and nutrition surveys of a nationally representative sample of non‐institutionalized adults conducted by the US National Center for Health Statistics (NCHS), Centers of Disease Control and Prevention. Specifically, we used data from the Third National Health and Nutrition Examination Survey (NHANES III, 1988–1994) and associated mortality data. Survey data including disease presence/absence, disease risk factors, and nutritional status were collected through household interviews, physical examinations, and blood and urine samples. Subjects were selected using a multistage stratified sampling method to ensure accurate representation of the US population. For more information on study sampling methods, see previous publications detailing the compilation of NHANES III.^[^ ^10^ ^]^

### Definitions of CLD

HBV was identified in individuals who tested positive for the hepatitis B surface antigen. Similarly, HCV was identified in individuals who tested positive for the HCV RNA. Participants were diagnosed with ALD if they had high serum aminotransferase levels (alanine transaminase levels [ALT] > 40 U/L or aspartate aminotransferase [AST] levels > 37 U/L in males, ALT or AST levels > 31 U/L in females) or signs of hepatic steatosis in those who reported excessive alcohol consumption (>20 g/day for males and >10 g/day for females) after excluding HCV, HBV, and other liver diseases.^[^ ^3^ ^]^ Participants who showed signs of hepatic steatosis and no other causes of CLD were determined to have NAFLD. Hepatic steatosis was identified by abdominal ultrasonography and graded as mild, moderate, or severe in individuals (ages 20–74).^[^ ^11^ ^]^ For the purpose of this study, participants with HBV, HCV, ALD, and NAFLD were categorized into the CLD cohort. Individuals without HBV, HCV, ALD, NAFLD, and any other type of CLD were categorized as the control cohort. Fibrosis‐4 index (FIB‐4) score for liver fibrosis was used to categorize patients with CLD into low fibrosis risk (FIB‐4 ≤ 2.67) and high fibrosis risk (FIB‐4 > 2.67).^[^ ^12^ ^]^

### Definitions of sarcopenia

Whole‐body bioimpedance analysis (BIA) measurement of resistance at 50 kHz was measured between the right wrist and ankle while in a supine position, using the Valhalla 1990B Bio‐Resistance Body Composition Analyzer (Valhalla Medical).

Skeletal muscle mass (SMM) was calculated using the following Janssen's formula,^[^ ^13^ ^]^ which had been validated using magnetic resonance imaging–measured skeletal muscle mass: skeletal mass (kg) = (height in cm)^2^/BIA resistance × 0.401 + (male × 3.825) + (age in years × −0.071) + 5.102, where BIA resistance is measured in ohms and male is coded for 1 for male and 0 for female. SMM was indexed to body mass (kg) to define skeletal muscle index (SMI).

Sarcopenia was defined using the European Working Group on Sarcopenia in Older People 2 recommendation on the use of normative (healthy young adult) with cutoff points at 2 SDs below the mean value.^[^ ^6^ ^]^ Individuals were diagnosed with sarcopenia if their SMI was higher than 1 SD below the gender‐specific mean for young adults (aged 20–39 years) in NHANES III (≥36.7% in men and ≥26.6% in female).

### Definitions of LS7 health metrics

The AHA's LS7 outlines three health factors (cholesterol level, BP, and blood glucose levels/glycemic control [GC]) and four health behaviors (BMI, physical activity [PA], smoking activity, and diet). We used these and revised definitions to obtain complete health metrics data from NHANES III.

The AHA‐proposed LS7 includes an established “ideal” status for each health metric. Meeting the ideal standard for each metric has been correlated with a lower all‐cause mortality.^[^ ^9^ ^]^ The three ideal health factors are defined as having an untreated cholesterol level < 200 mg/dl, a BP level < 120/80 mm Hg, and a GC of hemoglobin A1c < 5.7%. The four ideal health behaviors are defined as having a BMI of ≤ 25 kg/m^2^, participating in physical activity ≥5 times a week with metabolic equivalent tasks (METs) between 3 and 6, or ≥3 times a week with METs ≥ 6, self‐reporting as a current nonsmoker and having smoked < 100 cigarettes total, and a 2010 Healthy Eating Index (HEI) score of ≥69.3. The HEI included two subcategories: (1) dietary adequacy consisting of nine components (total vegetables, greens and beans, total fruits, whole fruits, whole grains, dairy, total protein foods, seafood and plant proteins, fatty acid ratio, and solid fats), with higher scores indicating higher consumption; and (2) dietary moderation with its three components (refined grains, sodium, and alcohols and added sugars), with higher scores indicating lower consumption. Poor diet is defined as HEI score < 56.9, intermediate diet as HEI score = 56.9–69.2, and ideal diet as HEI score ≥ 69.3. Definitions of ideal, intermediate, and poor LS7 metrics are displayed in Table S1. For each NHANES participant, a score of 1 was given if an ideal metric was met, and a score of 0 was given if an ideal metric was not met. Participant LS7 scores were added to give an overall LS7 health score.

### Mortality status determination

A follow‐up analysis led by the NCHS was conducted to determine the mortality status of NHANES III participants. All‐cause mortality data and mortality data caused by CVD, cancer, and diabetes were used in this study. Participant death due to major CVD and cerebrovascular diseases (International Classification of Diseases, Tenth Edition [ICD‐10] codes I00‐I90, I11, I13, I20‐I51, and I60‐I69) was classified as cardiovascular mortality.^[^ ^14^ ^]^ Similarly, patient death caused by malignancy or diabetes was classified as either cancer or diabetes mortality, respectively. For liver mortality, we assessed the restricted‐use Linked Mortality Files through the NCHS Research Data Center. Unlike the public‐use files, the restricted files provide ICD‐10 codes for underlying cause and contributory cause of death, which enabled us to identify liver‐specific death (Table S2). Liver‐specific death was identified by either CLD codes for underlying cause of death or CLD‐related complication codes for underlying cause of death, with CLD codes as a contributory cause of death. We used the published coding algorithm to overcome the underreported liver‐specific deaths.^[^ ^15^ ^]^

The NCHS conducted linkage and probabilistic record matching using National Death Index (NDI) data to determine participant mortality status.^[^ ^16^ ^]^ The NDI initially used ICD‐9 codes for mortality data from 1979–1998, and the NCHS recorded these data using the updated ICD‐10 codes. (See previously published literature for a more in‐depth comparison of ICD‐9 and ICD‐10 codes.^[^ ^17^ ^]^) The mortality status of all participants who were linked to the NDI was recorded in the NCHS follow‐up data. Participant length of survival was determined by the amount of time between the date of completion of the NHANES III survey to time of death or December 31, 2015, whichever came first. Participants who were not listed in mortality records were presumed as alive at the time of the follow‐up analysis.

### Additional data collection

Study data also included general information on race/ethnicity, income level, age, education, and medical history. Participant race and ethnicity were categorized into four groups: non‐Hispanic White, non‐Hispanic Black, Mexican American, or other. Income level was classified as low (poverty income rate [PIR] < 1.3), middle (PIR 1.3–3.5), and high (PIR > 3.5).^[^ ^18^ ^]^ We also noted participants' age (years), highest degree of education, and medical history.

### Statistical analysis

The baseline characteristics and LS7 metrics are reported as weighted prevalence values and SEM. Comparisons of covariates across individuals with different CLDs and by the presence of sarcopenia were performed using a t‐statistic for continuous variables and the Rao‐Scott chi‐square test for categorical variables.

Cox proportional hazards regression models were used to estimate hazard ratios (HRs) and their corresponding 95% confidence interval (CI) for each ideal LS7 metric and sarcopenia on all‐cause mortality. Multivariable models were constructed in several stages, including unadjusted, age‐sex adjusted, socio‐demographic adjusted, and important confounders–adjusted models. The full model was adjusted for age, sex, race/ethnicity, income, education, marital status, and ideal LS7 metrics. The proportional hazards assumption of the Cox models was examined by testing time‐dependent covariates,^[^ ^19^ ^]^ which showed no significant departure from proportionality over time. For cause‐specific mortality, we performed competitive risk analysis by treating all of the competing events as censored observations in the Cox model. Competing risks arise when a patient is at risk of more than one mutually exclusive event, such as death from a different cause, which will prevent any other from occurring. Subdistribution HRs by Fine‐Gray models were not performed because of the survey‐based design.

For subjects with NAFLD, regression models were further repeated stratified by sex, age, and BMI group (lean, overweight, and obese). Because of the small sample size and events, we reported only unadjusted and age‐sex adjusted HRs for cause‐specific mortality. We examined an interaction term of age, sex, and BMI group with sarcopenia for all‐cause and cause‐specific mortality in the Cox models, which none of them were significant (*p* > 0.05) (data not reported).

The effect of reaching each ideal LS7 metric on sarcopenia was evaluated by multivariable logistic regression among individuals with NAFLD.

All analyses were implemented by incorporating the sampling weights to obtain the nationally representative estimates. To account for the sampling design, sampling errors were estimated by the Taylor series linearization.^[^ ^20^ ^]^ As a result, the findings of the current study should be generalizable to the US population aged 20–74 years. Data were analyzed with SAS software, version 9.4 (SAS Institute), and statistical tests were considered significant at *p* < 0.05 (two tails).

## RESULTS

Of the 19,172 nonpregnant participants from NHANES III, 17,367 (90.6%) attended an examination at a mobile examination center. We excluded 3014 who were not eligible for an ultrasound examination due to age being older than 75 years or less than 20 years. An additional 2321 were also excluded for the following reasons: 872 had an ultrasound that was ungradable or missing; 799 had missing data for serum hepatitis B surface antigen; 872 had missing data for HCV RNA; 276 had insufficient data for the diagnosis of ALD; and 4 had HBV‐HCV coinfection. The final study cohort included 12032 NHANES participants.

### Sociodemographic and clinicodemographic characteristics of the final study cohort

In the final study cohort, prevalence of CLD was 34.9%, whereas controls constituted 65.1% of the study cohort. Among patients with CLD (34.9%), NAFLD was most prevalent (26.9%), followed by ALD (5.7%), HCV (1.8%), and HBV (0.5%). Most patients with NAFLD and ALD were White, while more than a fifth to a quarter of patients with HCV and HBV were Black. Details on sociodemographic and clinicodemographic features of the participants with CLDs and controls are presented in Table 1.

**TABLE 1:** Weighted prevalence of sarcopenia and Life's Simple 7 (LS7) metrics in subjects with CLD, NHANES III (1988–1994)

	NAFLD	HCV	HBV	ALD	Control
Age, mean (SEM)	46.01 (0.47)	39.49 (0.94)	41.12 (1.70)	43.92 (1.33)	41.56 (0.40)
Male, %	50.77 (1.08)	68.95 (4.49)	65.39 (6.76)	64.57 (3.38)	48.19 (0.75)
Race, %					
Non‐Hispanic White	75.28 (1.71)	56.03 (7.20)	48.29 (9.96)	81.11 (2.71)	77.54 (1.31)
Non‐Hispanic Black	9.01 (0.70)	21.67 (3.51)	25.54 (5.29)	7.99 (1.42)	10.45 (0.69)
Mexican American	6.60 (0.72)	6.29 (1.46)	1.83 (1.10)	6.15 (0.90)	4.58 (0.40)
Other race	9.11 (1.17)	16.00 (6.71)	24.35 (6.19)	4.74 (1.90)	7.43 (0.84)
Low income, %	17.67 (1.47)	41.77 (5.94)	32.53 (9.10)	17.30 (2.97)	16.67 (1.13)
College, %	37.33 (1.86)	19.62 (5.42)	39.70 (9.93)	41.57 (3.77)	45.47 (1.43)
Married, %	71.62 (1.46)	48.66 (4.66)	60.91 (10.37)	63.56 (4.71)	67.51 (0.92)
Sarcopenia	40.71 (1.80)	22.41 (4.10)	16.76 (5.63)	27.19 (3.75)	18.46 (0.97)
LS7 metric					
Total serum cholesterol, mg/dl					
Ideal (<200 untreated)	42.77 (1.81)	71.04 (4.87)	60.97 (9.94)	38.40 (3.27)	52.81 (1.11)
Intermediate (200–239 or treated)	35.33 (1.35)	23.84 (4.16)	29.83 (10.18)	33.85 (3.63)	30.31 (0.82)
Poor (≥240)	21.91 (1.17)	5.12 (2.06)	9.20 (4.44)	27.75 (3.13)	16.87 (0.69)
Glycemic control, HbA1c^a^					
Ideal (<5.7%)	71.12 (1.48)	74.08 (5.62)	86.42 (4.62)	87.69 (2.35)	86.04 (0.92)
Intermediate (5.7–6.4)	19.15 (1.08)	18.85 (5.59)	10.93 (3.93)	8.71 (2.17)	11.66 (0.84)
Poor (≥6.5%)	9.73 (0.77)	7.07 (2.36)	2.65 (2.30)	3.60 (0.95)	2.30 (0.24)
Smoking status					
Ideal	42.89 (1.38)	18.67 (5.48)	55.19 (8.05)	25.05 (3.89)	43.18 (1.13)
Intermediate	33.03 (1.51)	17.96 (6.21)	18.80 (9.96)	31.02 (4.33)	23.76 (0.76)
Poor	24.08 (1.13)	63.37 (6.74)	26.01 (7.60)	43.93 (3.60)	33.05 (0.98)
Blood pressure, mm Hg					
Ideal (<120/80 untreated)	36.89 (1.17)	43.71 (5.77)	64.11 (7.90)	33.11 (4.82)	52.57 (1.02)
Intermediate (120–129/80 or treated)	15.56 (0.80)	13.87 (2.70)	8.20 (4.03)	14.81 (2.72)	14.52 (0.69)
Poor (≥130/80)	47.55 (1.45)	42.42 (4.47)	27.69 (7.34)	52.08 (4.01)	32.91 (0.97)
Healthy diet score^b^					
Ideal (>69.3)	35.09 (1.42)	26.07 (7.50)	25.97 (6.47)	26.40 (3.49)	32.78 (1.16)
Intermediate (56.9–69.3)	33.10 (1.03)	33.94 (5.80)	50.44 (11.37)	35.18 (3.56)	35.09 (1.05)
Poor (<56.9)	31.80 (1.31)	39.99 (6.37)	23.59 (6.72)	38.42 (3.93)	32.13 (0.94)
Body mass index					
Ideal (<25)	27.30 (1.37)	55.44 (5.37)	66.08 (7.22)	32.17 (3.18)	52.67 (1.05)
Intermediate (25–29)	33.27 (1.24)	21.20 (3.55)	24.51 (6.28)	40.26 (3.64)	32.80 (0.68)
Poor (≥30)	39.43 (1.76)	23.36 (5.18)	9.41 (4.23)	27.57 (2.94)	14.53 (0.72)
Physical activity					
Ideal	37.48 (1.94)	40.29 (5.14)	50.01 (7.62)	38.56 (3.60)	42.27 (1.36)
Intermediate	47.17 (1.84)	41.06 (5.73)	27.08 (8.28)	47.30 (3.41)	45.91 (1.01)
Poor	15.35 (1.11)	18.66 (3.71)	22.91 (6.72)	14.14 (2.84)	11.82 (0.77)
No. of ideal LS7 metrics					
0	4.90 (0.43)	0.17 (0.18)	0	2.74 (0.76)	1.59 (0.19)
1	14.38 (1.18)	6.96 (2.23)	4.15 (2.83)	14.40 (3.07)	6.78 (0.45)
2	22.96 (1.04)	17.10 (3.69)	8.37 (2.53)	26.90 (4.58)	15.52 (0.68)
3	22.52 (1.03)	34.16 (4.03)	14.93 (5.53)	23.30 (3.30)	22.41 (0.77)
4	18.31 (1.17)	26.26 (5.70)	39.84 (11.81)	19.75 (3.14)	24.63 (0.74)
5	11.05 (1.03)	10.01 (3.52)	20.03 (7.09)	10.23 (2.37)	17.73 (0.77)
6	4.15 (0.49)	4.98 (2.56)	7.45 (3.91)	2.68 (1.30)	9.08 (0.68)
7	1.74 (0.32)	0.35 (0.25)	5.23 (4.63)	0	2.26 (0.35)
Elevated AST	7.57 (0.68)	52.06 (6.35)	26.05 (10.36)	22.76 (3.02)	2.54 (0.32)
Elevated ALT	9.88 (0.91)	45.70 (7.29)	24.05 (10.44)	21.31 (3.70)	2.94 (0.38)
Elevated liver enzyme	11.74 (0.81)	58.69 (5.63)	26.05 (10.36)	33.44 (3.87)	4.00 (0.40)
FIB‐4, %					
Severe risk, FIB‐4 ≥ 2.67	1.09 (0.18)	10.09 (2.48)	7.46 (4.65)	5.98 (1.88)	0.97 (0.14)
Moderate risk, FIB‐4 1.30–2.67	17.41 (0.99)	16.13 (3.03)	25.68 (9.27)	20.14 (3.82)	14.13 (0.74)
Low risk, FIB‐4 < 1.30	81.50 (0.99)	73.78 (4.65)	66.86 (9.54)	73.88 (3.77)	84.90 (0.78)
CKD	11.74 (0.71)	9.37 (2.80)	4.22 (2.09)	9.28 (2.25)	7.57 (0.33)
High risk for CVD	31.26 (0.95)	13.94 (3.35)	14.87 (5.40)	28.59 (3.54)	18.86 (0.88)
History of, %					
Cancer	6.57 (0.50)	7.05 (3.67)	1.18 (0.93)	6.58 (1.76)	6.11 (0.44)
CVD	6.21 (0.44)	4.73 (2.01)	0.40 (0.41)	4.16 (1.54)	3.21 (0.34)
Family CVD	18.16 (0.88)	22.53 (4.62)	11.40 (5.59)	18.56 (2.94)	16.75 (0.64)
Cumulative mortality, %					
All causes	29.55 (1.28)	35.18 (5.42)	22.62 (7.37)	34.73 (3.37)	20.74 (0.94)
CVD	7.33 (0.59)	5.72 (1.94)	0	7.71 (2.30)	5.25 (0.30)
Cancer	7.04 (0.72)	4.28 (2.00)	2.93 (1.23)	12.85 (2.71)	5.64 (0.37)
Diabetes	1.36 (0.27)	2.29 (1.35)	3.93 (3.81)	0.52 (0.30)	0.29 (0.09)

*Note*: All values are displayed as weighted percentages (95% confidence interval [CI]) except where otherwise noted. Mortality status followed up to December 2015.

Abbreviations: ALT, alanine aminotransferase; AST, aspartate aminotransferase; CKD, chronic kidney disease; FIB‐4, Fibrosis‐4 index; HbA1c, hemoglobin A1c.

^a^ Hemoglobin A1c values were used as proxies for blood glucose metric.

^b^ Healthy diet score was calculated based on the 2010 Healthy Eating Index recommended by Dietary Guidelines for Americans.

We assessed the prevalence of LS7 metrics based on different CLDs. Among different causes of CLD, patients with NAFLD had lower rates of ideal LS7 metrics. In this context, the lowest ideal GC rate was in NAFLD, followed by HCV, HBV, and ALD. Similarly, the lowest ideal BMI rate was noted among NAFLD at 27.3%, followed by ALD (32.2%), HCV (55.4%), and HBV (66.1%). Finally, the lowest ideal PA rate was noted among NAFLD at 37.5%, followed by ALD (38.6%), HCV (40.3%), and HBV (50%) (Table 1).

About 20% of patients with NAFLD had < 1 ideal metric, and a lower proportion of subjects with NAFLD met the ideal criteria for BMI, PA, BP control, GC, and serum total cholesterol as compared to those with HBV and HCV. In some categories, NAFLD and ALD had similar and relatively lower proportions of health metrics (Table 1).

### Sarcopenia, LS7 metrics, and types of CLD

**FIGURE 1:**
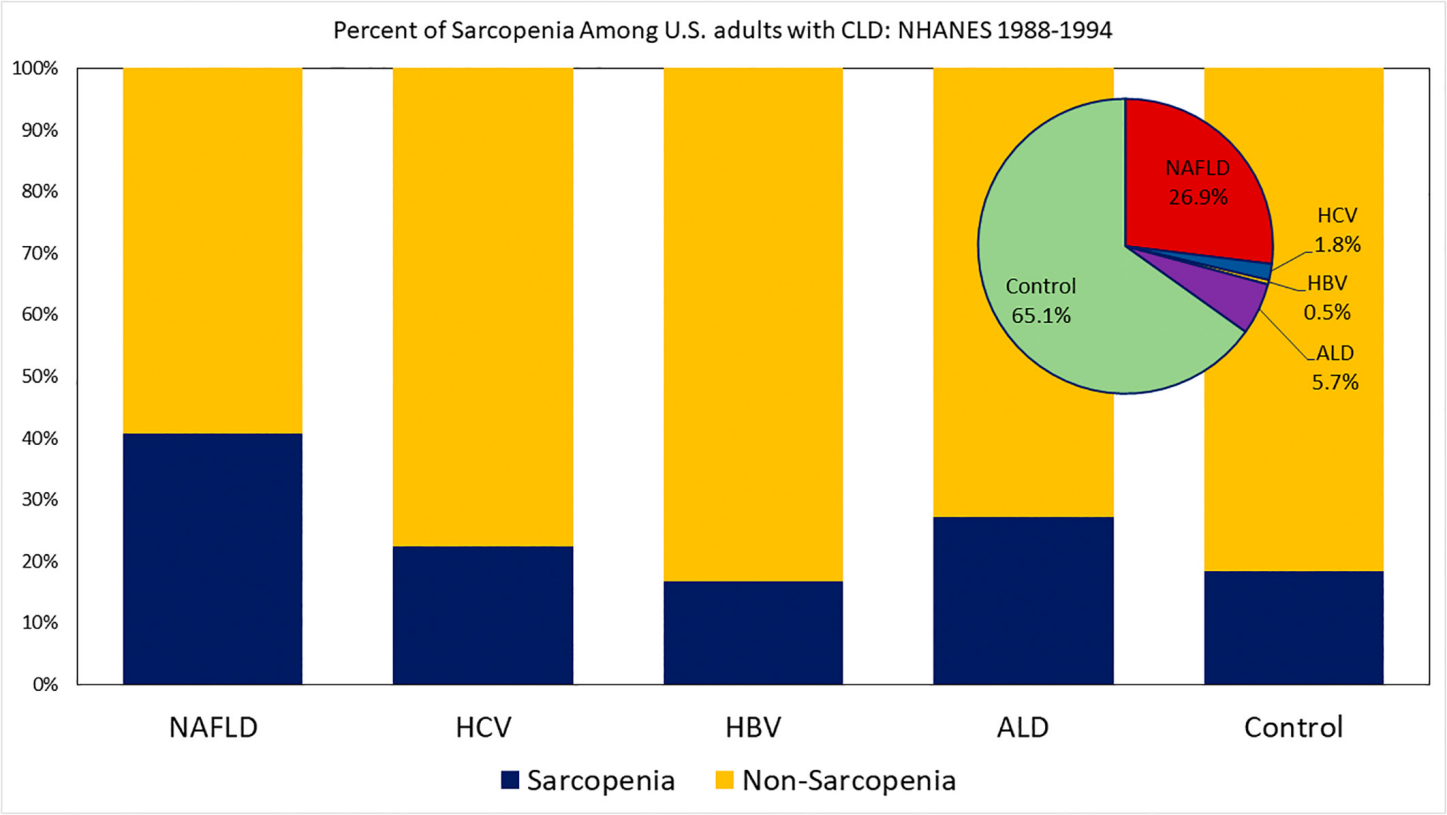
Percent of sarcopenia among US adults with chronic liver disease (CLD): Third National Health and Nutrition Examination Survey (NHANES III, 1988–1994). ALD, alcohol‐associated liver disease; HBV, hepatitis B virus; HCV, hepatitis C virus; NAFLD, nonalcoholic fatty liver disease.

Among the four CLD groups and controls, sarcopenia was most common in patients with NAFLD (40.7%), followed by ALD (27.2%), HCV (22.4%), controls (18.5%), and HBV (16.8%) (Figure 1 and Table 1).

The impact of the presence of sarcopenia on ideal LS7 metrics was most prominent in the groups with NAFLD and ALD as compared with other CLDs and controls (Table S3). Among patients with NAFLD, patients with sarcopenia had significantly worse profile for LS7 metrics than patients with NAFLD without sarcopenia. In patients with ALD, those with sarcopenia had significantly worse profiles in GC, BP, and BMI than patients with ALD without sarcopenia.

**FIGURE 2:**
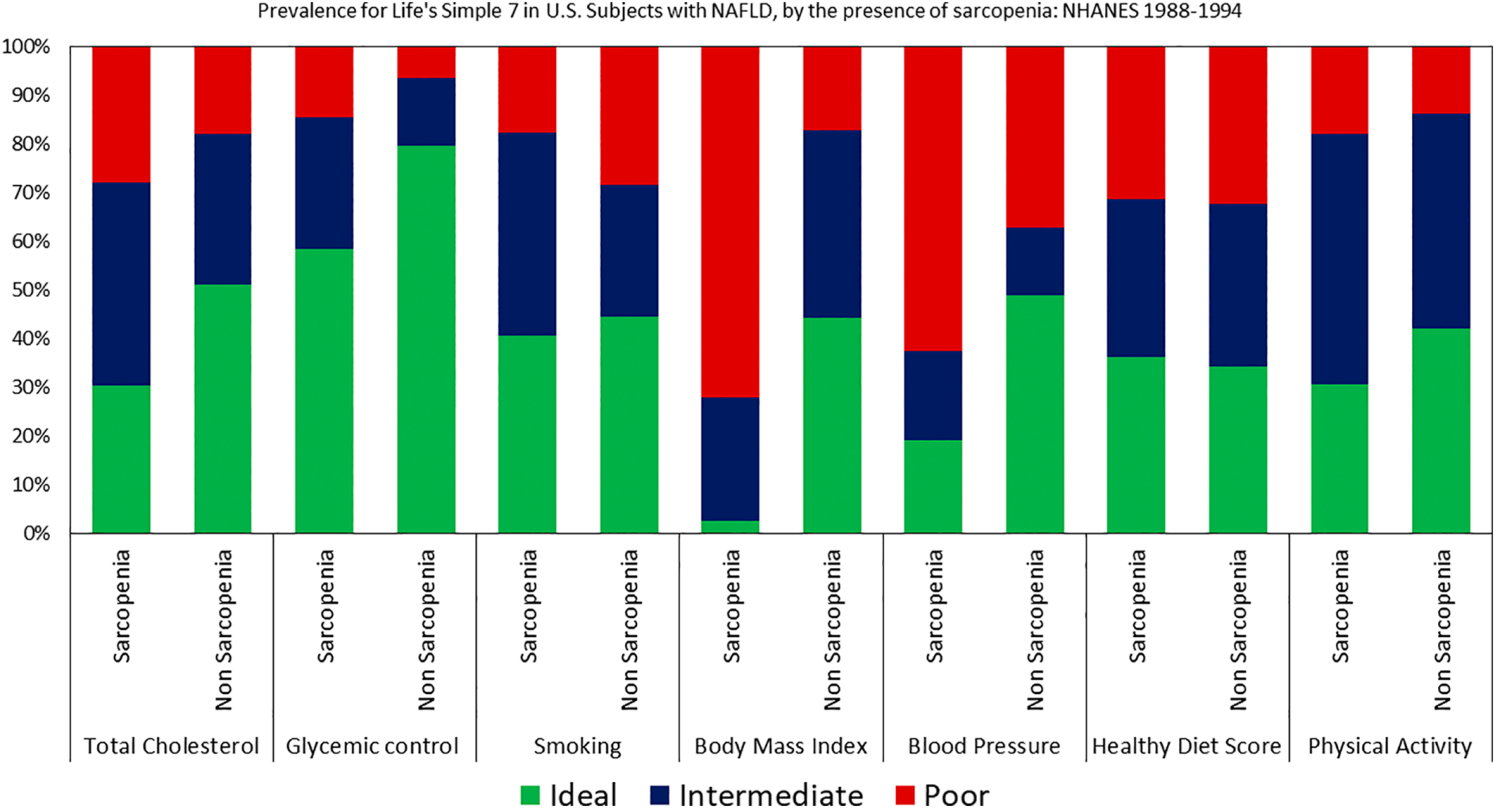
Prevalence for Life's Simple 7 in US adults with nonalcoholic fatty liver disease (NAFLD), by the presence of sarcopenia: NHANES III (1988–1994).

The only ideal LS7 metric that had higher prevalence among the group with sarcopenia than the group without sarcopenia was smoking (Figure 2 and Table S3). Additionally, the ideal LS7 metric related to diet was no different between subjects with sarcopenia and without sarcopenia.

### Sarcopenia and LS7 metrics for all‐cause mortality among subjects with CLD

During up to 27 years of follow‐up (median, 22.8 years; interquartile range, 20.4–24.8 years), among 4 patients with CLD and the controls, all‐cause cumulative mortality was highest among patients with HCV (35.2%), followed by ALD (34.7%), NAFLD (29.6%), HBV (22.6%), and the controls (20.7%) (Table 1).

In the unadjusted model, presence of sarcopenia was a higher risk of all‐cause mortality among patients with NAFLD (HR = 2.31, 95% CI 1.97–2.70; *p* < 0.01), ALD (HR = 2.40, 95% CI 1.73–3.35; *p* < 0.01), and the control group (HR = 2.44, 95% CI 2.03–2.92; *p* < 0.01). However, the age‐sex adjusted HR of sarcopenia for all‐cause mortality was significant only among subjects with NAFLD. Even after additional adjustments with the ideal level of LS7 metrics, the HR of sarcopenia remained significant among patients with NAFLD (HR = 1.24, 95% CI 1.01–1.54; *p* = 0.04) (Table 2).

**TABLE 2:** Hazard ratio of sarcopenia and LS7 metrics on all‐cause mortality among subjects with CLD: NHANES III (1988–1994)

	NAFLD		HCV		HBV		ALD		Control	
	HR (95% CI)	*p*	HR (95% CI)	*p*	HR (95% CI)	*p*	HR (95% CI)	*p*	HR (95% CI)	*p*
Unadjusted model										
Sarcopenia	2.31 (1.97–2.70)	<0.0001	2.23 (0.98–5.11)	0.0568	1.54 (0.48–4.99)	0.4613	2.40 (1.73–3.35)	<0.0001	2.44 (2.03–2.92)	<0.0001
Age‐sex adjusted model										
Sarcopenia	1.27 (1.1–1.46)	0.0015	1.14 (0.6–2.17)	0.6779	1.8 (0.6–5.41)	0.2915	1.36 (0.89–2.06)	0.1507	1.16 (0.98–1.38)	0.0831
Fully adjusted model										
Sarcopenia	1.24 (1.01–1.54)	0.0446	0.95 (0.43–2.09)	0.9035	1.00 (0.05–21.03)	0.9996	1.29 (0.76–2.19)	0.3338	1.04 (0.85–1.27)	0.7195
Age group										
Ages 20–39	Reference		Reference		Reference		Reference		Reference	
Ages 40–59	3.10 (2.03–4.75)	<0.0001	6.84 (3.33–14.05)	<0.0001	0.50 (0.08–3.07)	0.4495	3.61 (1.64–7.93)	0.0019	1.91 (0.43–8.53)	0.3913
Ages 60–75	14.98 (10.32–21.75)	<0.0001	13.07 (3.00–56.94)	0.001	0.67 (0.01–44.4)	0.8489	13.18 (5.7–30.49)	<0.0001	141.39 (66.06–302.62)	<0.0001
Male	1.31 (1.08–1.58)	0.0065	0.88 (0.35–2.23)	0.7803	0.41 (0.06–3.00)	0.3732	0.75 (0.37–1.55)	0.4326	1.26 (0.99–1.59)	0.0587
Race										
Non‐Hispanic White	Reference		Reference		Reference		Reference		Reference	
Non‐Hispanic Black	0.89 (0.71–1.12)	0.3226	1.1 (0.53–2.27)	0.7936	0.16 (0.01–2.49)	0.1854	1.61 (0.91–2.84)	0.101	0.9 (0.73–1.11)	0.3287
Mexican American	0.76 (0.62–0.93)	0.009	1.2 (0.44–3.31)	0.7158	0.31 (0.01–13.17)	0.5298	0.84 (0.44–1.58)	0.5813	1.01 (0.72–1.43)	0.9354
Other race	0.35 (0.19–0.63)	0.0007	1.6 (0.42–6.09)	0.4833	1.71 (0.13–22.69)	0.6792	0.66 (0.21–2.11)	0.4756	0.82 (0.57–1.17)	0.2705
Low income	1.43 (1.11–1.85)	0.0072	1.04 (0.52–2.08)	0.9109	0.49 (0.04–5.75)	0.5665	2.36 (1.27–4.38)	0.0076	1.63 (1.29–2.05)	0.0001
College	0.74 (0.59–0.94)	0.0136	0.59 (0.22–1.57)	0.2826	0.45 (0.11–1.87)	0.2644	0.73 (0.42–1.3)	0.2794	0.79 (0.64–0.97)	0.0284
Married	0.86 (0.69–1.08)	0.1844	0.84 (0.37–1.91)	0.6709	1.00 (0.32–3.11)	0.9967	0.65 (0.38–1.12)	0.1162	0.86 (0.69–1.08)	0.1825
Total serum cholesterol, ideal level	0.98 (0.80–1.21)	0.8617	0.98 (0.44–2.19)	0.9569	47.59 (1.31–1728.33)	0.0356	0.77 (0.41–1.42)	0.3859	1.07 (0.84–1.38)	0.5757
Glycemic control, ideal level	0.60 (0.50–0.72)	<0.0001	0.89 (0.47–1.7)	0.7164	0.18 (0.05–0.68)	0.0119	1.04 (0.63–1.73)	0.8802	0.40 (0.33–0.49)	<0.0001
Smoking status, ideal level	0.60 (0.50–0.72)	<0.0001	0.6 (0.23–1.58)	0.2925	0.34 (0.10–1.21)	0.094	0.39 (0.21–0.73)	0.0037	0.63 (0.51–0.78)	<0.0001
Blood pressure, ideal level	0.76 (0.60–0.96)	0.0245	0.46 (0.21–0.98)	0.0443	1.82 (0.27–12.25)	0.5336	0.59 (0.3–1.17)	0.1269	0.74 (0.58–0.93)	0.0125
Body mass index, ideal level	1.30 (1.01–1.67)	0.0387	0.51 (0.25–1.03)	0.0607	0.14 (0.03–0.69)	0.0162	1.35 (0.72–2.53)	0.3426	1.08 (0.88–1.31)	0.4602
Healthy diet score, ideal level	0.95 (0.81–1.11)	0.5259	0.74 (0.38–1.43)	0.3594	0.28 (0.01–5.59)	0.3993	1.2 (0.59–2.43)	0.6047	0.81 (0.66–0.98)	0.0306
Physical activity, ideal level	0.83 (0.66–1.04)	0.0958	0.77 (0.48–1.25)	0.285	4.84 (1.14–20.58)	0.0336	0.93 (0.53–1.62)	0.7844	0.92 (0.79–1.07)	0.2766

Abbreviations: CI, confidence interval; HR, hazard ratio.

Among subjects with NAFLD, ideal GC, smoking status (SS), and BP offered significant protection against premature all‐cause deaths (HR = 0.60 [0.50–0.72; *p* < 0.01], 0.60 [0.50–0.72; <0.01], and 0.76 [0.60–0.96, *p* = 0.02], respectively). Furthermore, the health metric components with the strongest protection against all‐cause mortality were ideal level of BP among subjects with HCV; ideal level of GC and ideal BMI among those with HBV; ideal level of SS among those with ALD; and ideal levels of GC, SS, BP, and healthy diet score among the control group (Table 2).

In the sensitivity analysis, when sarcopenia was defined as SMI higher than 2 SDs below the gender‐specific mean for young adults, the prevalence of sarcopenia remained higher among NAFLD as compared with other CLDs and controls (6.37% in NAFLD, 4.51%% in ALD, 6.37% in HCV, 3.89% in HBV, and 1.16% in controls; *p* < 0.001). The unadjusted hazard ratio of sarcopenia by the new cutoff for all‐cause mortality among NAFLD (HR = 2.06, 95% CI 1.57–2.71; *p* < 0.001) and ALD (HR = 2.55, 95% CI 1.10–5.89; *p* = 0.03) remained significant. However, in the fully adjusted model, sarcopenia was no longer an independent predictor of all‐cause mortality among patients with NAFLD (HR = 1.18, 95% CI 0.90–1.54; *p* = 0.225), while it remained a predictor of all‐cause mortality among patients with ALD (HR = 2.33, 95% CI 1.14–4.77; *p* = 0.022). This suggests that the association between sarcopenia and all‐cause mortality of subjects with NAFLD was mediated by meeting the ideal level of LS7 metrics (data not shown).

### Competing risk analyses of sarcopenia for cause‐specific mortality among subjects with CLD

Competing risk analyses are summarized in Table 3. Crude HR showed that presence of sarcopenia was a higher risk for cardiovascular‐specific (HR = 2.28 [1.71–3.05]; *p* < 0.01), cancer‐specific (HR = 1.90 [1.37–2.65]; *p* < 0.01), diabetes‐specific (HR = 6.42 [2.87–14.36]; *p* < 0.01), and liver‐specific mortality (HR = 2.49 [1.08–5.76]; *p* = 0.04), among subjects with NAFLD. Additionally, sarcopenia posed a higher risk for cancer‐specific mortality among those with HBV and ALD, while no significant higher risk of cause‐specific mortality was noted among those with HCV. The age‐sex adjusted hazard ratios of sarcopenia remained significant for diabetes mortality among subjects with NAFLD and for cancer mortality among those with HBV.

**TABLE 3:** Competing risk analysis of sarcopenia on cause‐specific mortality among subjects with CLD: NHANES III (1988–1994)

	Unadjusted		Age‐sex adjusted^a^	
Subgroup	HR (95% CI)	*p*	HR (95% CI)	*p*
CVD mortality				
Control	2.59 (1.95–3.44)	<0.0001	1.08 (0.83–1.41)	0.5672
NAFLD	2.28 (1.71–3.05)	<0.0001	1.09 (0.81–1.48)	0.5620
HCV	0.10 (0.01–1.08)	0.0572	0.05 (0.01–0.47)	0.0102
HBV	NA		NA	
ALD	2.00 (0.83–4.83)	0.1188	1.43 (0.57–3.56)	0.4380
Cancer mortality				
Control	1.97 (1.43–2.73)	0.0001	1.01 (0.73–1.40)	0.9502
NAFLD	1.90 (1.37–2.65)	0.0003	1.11 (0.79–1.57)	0.5411
HCV	1.37 (0.28–6.78)	0.6958	0.63 (0.17–2.41)	0.4952
HBV	21.93 (2.05–234.13)	0.0117	28.02 (2.20–356.54)	0.0113
ALD	2.02 (1.03–3.99)	0.0423	1.44 (0.69–2.98)	0.3260
Diabetes mortality				
Control	2.42 (1.13–5.18)	0.0242	1.21 (0.51–2.90)	0.6575
NAFLD	6.42 (2.87–14.36)	<0.0001	3.76 (1.64–8.61)	0.0024
HCV	1.10 (0.15–8.23)	0.9226	0.46 (0.08–2.52)	0.3634
HBV	NA		NA	
ALD	1.93 (0.30–12.39)	0.4809	0.91 (0.10–8.16)	0.9281

*Note*: Not applicable (NA) because of a small size of event (<5) or the nonconverged model.

^a^ Model adjusted for age, sex, race, income, education, married, and ideal level of LS7 metrics (total serum cholesterol, glycemic control, smoking status, blood pressure, body mass index, healthy diet score, physical activity).

Among subjects with NAFLD, we conducted additional stratified analyses (sex, age group, and BMI group) to assess the impact of sarcopenia on all‐cause and cause‐specific mortality (Table 4). Subgroup analyses of subjects with NAFLD revealed that all‐cause mortality risk associated with sarcopenia in age‐sex adjusted model was significant in males (HR = 1.27 [1.02–1.59]; *p* = 0.04), females (HR = 1.27 [1.02–1.60]; *p* = 0.04), and subjects with lean NAFLD (BMI = 18.5–24.9; HR = 2.31 [1.39–3.84]; *p* < 0.01). Competing risk analyses also revealed that cancer‐specific mortality associated with sarcopenia was significant in subjects with lean NAFLD (HR = 3.07 [1.04–9.08]; *p* = 0.04). Finally, sarcopenia was associated with liver‐specific mortality among females with NAFLD (HR = 2.53 [1.23–5.21]; *p* = 0.01).

**TABLE 4:** Hazard ratio of sarcopenia for all‐cause and cause‐specific mortality among subjects with NAFLD, stratified by sex, age group, and BMI group: NHANES III, 1988–1994

	Unadjusted		Age‐sex adjusted	
Subgroup	HR (95% CI)	*p*	HR (95% CI)	*p*
All‐cause mortality				
Sex				
Female	2.67 (2.14–3.33)	<0.0001	1.27 (1.02–1.60)	0.0362
Male	2.13 (1.68–2.71)	<0.0001	1.27 (1.02–1.59)	0.0355
Age group (years)				
Ages 20–39	2.59 (0.49–13.81)	0.2591	1.94 (0.88–4.29)	0.1011
Ages 40–59	1.34 (0.65–2.76)	0.4167	NA	
Ages 60–75	0.94 (0.64–1.37)	0.7467	1.11 (0.94–1.32)	0.2011
BMI group				
BMI 18.5–24.9	8.16 (5.14–12.97)	<0.0001	2.31 (1.39–3.84)	0.0017
BMI 25.0–29.9	2.63 (1.83–3.78)	<0.0001	1.16 (0.86–1.57)	0.3210
BMI ≥ 30.0	1.84 (1.33–2.56)	0.0004	1.06 (0.81–1.40)	0.6565
CVD mortality				
Sex				
Female	3.15 (2.02–4.91)	<0.0001	1.41 (0.95–2.09)	0.0852
Male	1.74 (1.10–2.76)	0.0193	0.91 (0.58–1.44)	0.6825
Age group (years)				
Ages 20–39	2.59 (0.49–13.81)	0.2591	2.58 (0.49–13.68)	0.2592
Ages 40–59	1.34 (0.65–2.76)	0.4167	1.34 (0.65–2.78)	0.4191
Ages 60–75	0.94 (0.64–1.37)	0.7467	0.99 (0.68–1.44)	0.9400
BMI group				
BMI 18.5–24.9	4.4 (1.84–10.49)	0.0013	0.98 (0.48–1.97)	0.9457
BMI 25.0‐29.9	2.08 (1.10–3.94)	0.0249	0.82 (0.46–1.46)	0.4859
BMI ≥ 30.0	1.85 (1.03–3.33)	0.0407	0.93 (0.48–1.80)	0.8191
Cancer mortality				
Sex				
Female	1.97 (1.18–3.29)	0.0104	1.19 (0.69–2.05)	0.5278
Male	1.83 (1.07–3.14)	0.0288	1.09 (0.65–1.84)	0.7467
Age group				
Ages 20–39	1.08 (0.34–3.42)	0.8944	1.07 (0.34–3.39)	0.9024
Ages 40–59	1.22 (0.71–2.08)	0.4599	NA	
Ages 60–75	1.00 (0.62–1.60)	0.9945	1.06 (0.66–1.70)	0.8196
BMI group				
BMI 18.5–24.9	8.61 (3.23–22.92)	<0.0001	3.07 (1.04–9.08)	0.0429
BMI 25.0–29.9	2.06 (1.03–4.11)	0.0413	1.09 (0.56–2.11)	0.8032
BMI ≥ 30.0	1.62 (0.85–3.08)	0.1411	1.01 (0.53–1.93)	0.9744
Diabetes mortality				
Sex				
Female	4.04 (1.46–11.16)	0.0080	1.89 (0.72–4.93)	0.1893
Male	12.23 (3.32–45.09)	0.0003	8.08 (2.05–31.89)	0.0036
Age group (years)				
Ages 20–39	3.44 (0.79–14.98)	0.0984	3.48 (0.80–15.12)	0.0949
Ages 40–59	6.44 (1.79–23.17)	0.0052	6.37 (1.77–22.97)	0.0055
Ages 60–75	1.83 (0.64–5.26)	0.2568	1.67 (0.57–4.83)	0.3405
BMI group				
BMI 18.5–24.9	NA		NA	
BMI 25.0–29.9	NA		1.18 (0.24–5.69)	0.8372
BMI ≥ 30.0	3.34 (1.05–10.63)	0.0413	2.23 (0.70–7.09)	0.1695
Liver mortality				
Sex				
Female	4.43 (2.06–9.50)	0.0003	2.53 (1.23–5.21)	0.0126
Male	1.40 (0.33–6.01)	0.6429	1.19 (0.30–4.70)	0.7956
Age group (years)				
Ages 20–39	4.44 (0.79–25.03)	0.0892	4.37 (0.81–23.61)	0.0852
Ages 40–59	1.46 (0.65–3.25)	0.3523	1.53 (0.63–3.70)	0.3433
Ages 60–75	1.45 (0.55–3.85)	0.4439	1.24 (0.49–3.10)	0.6429
BMI group				
BMI 18.5–24.9	NA		NA	
BMI 25.0‐29.9	1.76 (0.34–9.19)	0.4944	1.44 (0.45–4.67)	0.5343
BMI ≥ 30.0	1.89 (0.61–5.83)	0.2626	1.43 (0.39–5.26)	0.5796

*Note*: Competing risk analysis was performed for cause‐specific analysis.

Abbreviations: BMI, body mass index; NA, not applicable because of a small size of event (<5) or the nonconverged model.

### Associations between LS7 metrics and sarcopenia among subjects with NAFLD

To evaluate the associations of LS7 metrics and sarcopenia among subjects with NAFLD, logistic regression analyses were carried out (Table 5). In our fully adjusted model, components of the LS7 metrics with the strongest protection against sarcopenia in NAFLD included ideal BMI (odds ratio [OR]: 0.04 [95% CI: 0.03–0.06], *p* < 0.01), ideal BP (OR = 0.60 [0.40–0.89], *p* = 0.01), ideal PA (OR = 0.67 [0.51–0.87], *p* = 0.03), and ideal GC (OR = 0.74 [0.56–0.97], *p* = 0.03). In the same model, compared with subjects aged 20–39, those aged 40–59 and aged 60–75 years had 63%–250% higher risk of sarcopenia (OR = 1.63 [1.07–2.48], *p* = 0.02; and OR = 3.50 [2.15–5.70], *p* < 0.01; respectively). Additionally, as compared with male, female had +43% higher risk of having sarcopenia (OR = 1.59 [1.10–2.29], *p* = 0.01).

**TABLE 5:** Odd ratio of ideal LS7 metrics for sarcopenia among subjects with NAFLD: NHANES III (1988–1994)

	OR (95% CI)	*p*		OR (95% CI)	*p*
Age group (years)			Age group (years)		
20–39	Reference		20–39	Reference	
40–59	1.63 (1.07–2.48)	0.0231	40–59	1.68 (1.16–2.42)	0.007
60–75	3.50 (2.15–5.70)	<0.0001	60–75	3.07 (2.12–4.46)	<0.0001
Female	1.59 (1.10–2.29)	0.0138	Male	1.43 (1.03–1.99)	0.032
Race			Race		
Non‐Hispanic White	Reference		Non‐Hispanic White	Reference	
Non‐Hispanic Black	1.33 (0.90–1.97)	0.1504	Non‐Hispanic Black	1.29 (0.90–1.84)	0.1582
Mexican American	0.95 (0.71–1.28)	0.7438	Mexican American	1.10 (0.82–1.48)	0.5262
Other race	0.72 (0.43–1.20)	0.2014	Other race	0.74 (0.47–1.18)	0.2025
Low income	0.85 (0.63–1.16)	0.3097	Low income	0.89 (0.67–1.19)	0.4313
College	1.11 (0.83–1.47)	0.4857	College	1.08 (0.84–1.39)	0.5374
Married	0.87 (0.67–1.12)	0.2685	Married	0.99 (0.79–1.25)	0.9379
Ideal level of LS7 metrics			No. of ideal LS7 metrics		
Total serum cholesterol, ideal level	0.85 (0.66–1.09)	0.1873	0–1	Reference	
Glycemic control, ideal level	0.74 (0.56–0.97)	0.0276	2	0.87 (0.60–1.25)	0.4399
Smoking status, ideal level	0.95 (0.74–1.21)	0.6604	3	0.60 (0.40–0.88)	0.0105
Blood pressure, ideal level	0.60 (0.40–0.89)	0.0117	4	0.27 (0.16–0.44)	<0.0001
Body mass index, ideal level	0.04 (0.03–0.06)	<0.0001	≥5	0.05 (0.03–0.09)	<0.0001
Healthy diet score, ideal level	0.90 (0.66–1.22)	0.4703			
Physical activity, ideal level	0.67 (0.51–0.87)	0.0034			

*Note*: Full adjustments: age, male, race, income, education, marital status, and LS7 metrics.

As the number of ideal LS7 metrics increased, the risk of sarcopenia progressively decreased. In this context, we observed 40% reduction risk of sarcopenia for three ideal metrics (OR: 0.60 [0.40–0.88], *p* = 0.01) to 95% reduction for five or more ideal metrics (OR = 0.05 [0.03–0.09], *p* < 0.01) among subjects with NAFLD.

## DISCUSSION

CLD is a global health problem with significant clinical and economic burden. In the last decades, NAFLD has become a major driver of the global burden of liver disease, leading to increasing rates of cirrhosis, hepatocellular carcinoma, and liver transplantation.^[^ ^21^, ^22^, ^23^, ^24^, ^25^ ^]^ In addition to NAFLD, ALD, HCV and HBV are also contributing to the global burden of CLD.^[^ ^26^, ^27^ ^]^ Our current study provides an insight about the potential association among different types of CLDs with sarcopenia, modifiable risk factors (LS7), and mortality.

One of the most important findings of the current study is to describe the prevalence of healthy living as documented by LS7 metrics among the most common types of CLD. Our data showed that compared to patients with viral hepatitis, those with NAFLD and ALD had worse LS7 metric profiles, especially in GC, serum cholesterol level, BP control, and BMI. For patients with NAFLD, this can be explained by the close relationship between NAFLD and metabolic abnormalities.^[^ ^28^, ^29^ ^]^ In this context, it is widely accepted that NAFLD is the liver manifestation of metabolic syndrome, and patients with NAFLD have higher prevalence of insulin resistance, hypertension, hyperlipidemia, and obesity.^[^ ^30^, ^31^ ^]^ In fact, because of this close association, cardiovascular‐related mortality has been shown to be the leading cause of death among patients with NAFLD.^[^ ^32^ ^]^ Similar to patients with NAFLD, patients with ALD also had poor LS7 metrics. This finding is consistent with the current knowledge that ALD is also associated with hypertension and cardiovascular diseases.^[^ ^33^ ^]^ On the other hand, the LS7 profiles of patients with viral hepatitis–related CLD were similar to the controls, suggesting possibly milder impact by these metabolic abnormalities among these patients than those with NAFLD and ALD.

Another important finding of this study was the prevalence of sarcopenia among patients with four common causes of CLD. Additionally, we assessed the impact of sarcopenia on long‐term mortality of patients with CLD. Among the four leading causes of CLD, sarcopenia was most prevalent in patients with NAFLD. More importantly, among patients with NAFLD, the presence of sarcopenia was associated with poor LS7 metrics profile. This finding is in agreement with previous reports demonstrating the role of insulin resistance and metabolic syndrome components in the pathogenesis of sarcopenia among patients with cirrhosis and NAFLD, emphasizing the shared mediators and pathways between NAFLD and sarcopenia.^[^ ^34^, ^35^, ^36^, ^37^, ^38^, ^39^, ^40^ ^]^ Consistent with our findings that links LS7 metrics to sarcopenia among patients with CLD, a recent study from Korea reported significant associations among NAFLD, sarcopenia, and atherosclerotic cardiovascular disease.^[^ ^41^ ^]^ In that study, NAFLD and sarcopenia were significantly associated with increased risk of atherosclerotic cardiovascular disease. Although the mechanism of sarcopenia in ALD is similar to those with NAFLD, the direct impact of ethanol on skeletal muscle, along with its impact on the brain, could also contribute to muscle loss.^[^ ^42^ ^]^ In addition to causes of liver disease, severity of liver disease is a well‐known risk for sarcopenia.^[^ ^43^, ^44^, ^45^, ^46^ ^]^

One of the important aspects of the current study is to provide competing risk analyses of sarcopenia for cause‐specific mortality among patients with CLD. In this context, our data suggest that the long‐term outcome of patients with NAFLD is disproportionately impacted by presence of sarcopenia. In fact, presence of sarcopenia led to higher cardiovascular‐specific, cancer‐specific, diabetes‐specific, and liver‐specific mortality. Furthermore, our subgroup analyses for NAFLD demonstrated that the impact of sarcopenia was significant in all‐cause mortality and diabetes‐specific mortality among males, while sarcopenia was associated with all‐cause mortality and liver‐specific mortality among females. Considering the various sociodemographic and clinicodemographic features of different ethnic and cultural populations in the United States, as well as their varying risk factors for NAFLD, understanding gender‐specific risk of mortality in the presence of sarcopenia potentially has important clinical value. Therefore, clinical assessment of sarcopenia among patients with NAFLD should be considered in the clinical practice.

The main strength of the current study comes from using a nationally representative sample of a US multiethnic population with consideration of a multitude of potential confounding factors and long‐term follow‐up for mortality, which facilitates the generalization of the findings. We performed competing risk analysis of cause‐specific mortality using a nationally representative sample of the US population and evaluated liver mortality associated with sarcopenia by using the restricted‐use Linked Mortality Files through the NCHS Research Data Center. There are a few limitations to this study. First, the NHANES survey data lack liver histology data for NAFLD. Although NHANES III data were collected nearly 30 years ago, these are the only data with ultrasonographic data among NHANES survey cycles. The diagnosis of NAFLD was based on the detection of hepatic steatosis on liver ultrasound, which has known limitations for NAFLD diagnosis, especially when hepatic steatosis is less than 20%^[^ ^47^ ^]^ and has intra‐observer and interobserver variability. However, recent clinical practice guidelines recommend ultrasonography for first‐line imaging modality to define NAFLD, and NHANES III data reported the interrater reliability between readers at 88.7% agreement. We believe that the benefits of ultrasonography suitable for a large population‐based study would balance these limitations. Second, as NHANES included data from patient surveys and health behavior components of LS7 metrics (e.g., smoking activity, diet, physical activity) are heavily dependent on patient reports, reliability of the data might be questioned. Due to a cross‐sectional design of NHANES, the temporal causality of the observed association between LS7 metrics and sarcopenia could not be established. The interpretation of results from our logistic regression needs to be carefully understood, as reverse causality could be possible. Third, we used the predicted skeletal muscle mass using the BIA method, which could lead to misclassification. However, the high degree of accuracy (r = 0.93) between muscle mass predicted using BIA and those measured using magnetic resonance imaging was reported. In addition, our findings were relatively robust against the different cutoff value of SMI to define sarcopenia. Finally, we were unable to capture the effects of changes in LS7 metrics, sarcopenia, and liver disease that may have occurred over time because of lack of follow‐up data.

## CONCLUSIONS

NAFLD and ALD are the CLDs with poor healthy living, as documented by suboptimal LS7 metric scores. Both NAFLD and ALD are also associated with sarcopenia. Presence of sarcopenia among patients with NAFLD has a negative effect on mortality. In this context, assessment for sarcopenia among patients with NAFLD in the clinical practice should be strongly considered. Future studies need to assess other potential analyses of interaction of sarcopenia in the context of superimposed metabolic risks as well as its impact on the surrogate of long‐term outcomes (such as FIB‐4) in other liver diseases.

## CONFLICT OF INTEREST

Dr. Younossi is a consultant to BMS, Gilead, AbbVie, Novo Nordisk, Merck, Quest Diagnostics, Viking, Tern Pharmaceuticals, Siemens, and Intercept.

## Supporting information


**Table S1** Definitions of ideal, intermediate, and poor American Heart Association’s Life’s Simple 7
Table S2 List of International Classification of Diseases, Tenth Edition codes for cause‐specific deaths
Table S3 Weighted prevalence of sarcopenia and Life’s Simple 7 metrics in adults with chronic liver disease, by the presence of sarcopenia, Third National Health and Nutrition Examination Survey (1988–1994), stratified by different liver diseases

## Data Availability

https://wwwn.cdc.gov/nchs/nhanes/nhanes3/default.aspx
