# Supplementary material for: Sarcopenia, healthy living, and mortality in patients with chronic liver diseases
Source: Hepatol Commun. 2022 Aug 10;6(11):3140–53. doi: 10.1002/hep4.2061 (PMC9592796; doi:10.1002/hep4.2061)
Supplement: Supplementary file 1 — Table S1 Definitions of ideal, intermediate, and poor American Heart Association’s Life’s Simple 7 Table S2 List of International Classification of Diseases, Tenth Edition codes for cause‐specific deaths Table S3 Weighted prevalence of sarcopenia and Life’s Simple 7 metrics in adults with chronic liver disease, by the presence of sarcopenia, Third National Health and Nutrition Examination Survey (1988–1994), stratified by different liver diseases [file HEP4-6-3140-s001.docx]

| Supplementary Table 1. Definitions of Ideal, Intermediate, and Poor AHA’s Life’s Simple 7 | | |
| --- | --- | --- |
| Life’s Simple 7 Health Metrics | AHA definitions | Modified Definitions in NHANES III (1988-1994) |
| Body Mass Index |  |  |
| Ideal | <25 kg/m2 | <25 kg/m2 |
| Intermediate | 25–29.9 kg/m2 | 25–29.9 kg/m2 |
| Poor | ≥30 kg/m2 | ≥30 kg/m2 |
| Smoking Status |  |  |
| Ideal | Never or quit >12 months | Never; Since there is no information about the time since quitting in NHANES III, we used self-reported never smoker. |
| Intermediate | Former ≤ 12 months | Former reported |
| Poor | Current | Current reported |
| Physical Activity |  |  |
| Ideal | ≥150 min/wk moderate or ≥75 min/wk vigorous  or ≥150 min/wk moderate and vigorous together | Since there are no information about the duration of physical activity in NHANES III, we used physical activities with 3≤ metabolic equivalent tasks (METs)< 6 and ≥5 times/week or physical activities with ≥6 METs and ≥3 times/week. |
| Intermediate | 1–149 min/wk moderate  or 1–74 min/wk vigorous  or 1–149 min/wk moderate and  vigorous together | the difference between Ideal and Poor |
| Poor | None | No physical activity reported |
| Healthy Diet Score |  |  |
| Ideal | 4–5 Components  AHA’s healthy diet score is calculated from the sum of the following 5 components, one point each for the consumption of fruits and vegetables (>4.5 cups/day), fish (>two 3.5- oz servings/week), fiber-rich whole grains (> three 1-oz-equivalent servings/day), sodium (<1500 mg/d), and sugar-sweetened beverages (<36-oz/week). | The healthy eating index (HEI) was calculated based on the HEI-2010. HEI-2010 comprises 2 subcategories: 1) dietary adequacy composed of nine components [total vegetables (TV), greens and beans (GB), total fruits (TF), whole fruits (WF), whole grains (WG), dairy, total protein foods (TPF), seafood and plant proteins (SPP), fatty acid ratio (FAR), and solid fats (SF)] - higher scores indicated higher consumption and 2) dietary moderation with its three components [refined grains (RG), sodium, alcohols and added sugars (SoFAAs)] with higher scores indicating lower consumption.  HEI ≥ 69.3 |
| Intermediate | 2–3 Components | HEI 56.9-69.2 |
| Poor | 0–1 Components | HEI <56.9 |
| Blood Pressure, mm Hg |  |  |
| Ideal | <120 mmHg/ <80 mmHg and untreated | <120 mmHg/ <80 mmHg and untreated |
| Intermediate | SBP 120-139 mmHg or DBP 80–89 mmHg or treated to goal | SBP 120-139 mmHg or DBP 80–89 mmHg or treated to goal |
| Poor | SBP ≥140 mm Hg  or DBP ≥90 mm Hg | SBP ≥140 mm Hg  or DBP ≥90 mm Hg |
| Total Serum Cholesterol, mg/dL |  |  |
| Ideal | <200 mg/dL and untreated | <200 mg/dL and untreated |
| Intermediate | 200-239 mg/dL or treated to goal | 200-239 mg/dL or treated to goal |
| Poor | ≥240 mg/dL | ≥240 mg/dL |
| Glycemic control, HbA1c, % |  |  |
| Ideal | Fasting Blood Glucose <100 mg/dL | Since fasting glucose was only available for a sub-sample of participants in NHANES III (n=6,939), we used Hemoglobin A1c < 5.7%. |
| Intermediate | Fasting Blood Glucose 100-125 mg/dL | Hemoglobin A1c 5.7 - 6.4% |
| Poor | Fasting Blood Glucose ≥126 mg/dL | Hemoglobin A1c ≥ 6.4% |

| Supplementary Table 2. List of ICD 10 codes for Cause-specific Deaths | |
| --- | --- |
|  | ICD 10 CODE |
| **Cardiovascular disease** | I00-I78 |
| **Cancer** | C00-C97 |
| **Diabetes** | E10-E14 |
| **Chronic liver disease** | |
| Acute and subacute necrosis of liver | K72.0x, K76.2 |
| Chronic liver disease and cirrhosis | K70.xx, K72.1x, K73.x, K74.xx, K75.4, K75.8x, K76.0, K76.89, K76.9 |
| Liver abscess and sequelae of chronic liver disease | K72.9x, K75.0-1, K76.6-7 |
| Malignant neoplasm of liver and intrahepatic bile duct | C22.0-2, C22.7-9 |
| Other disorders of the liver | K71.0-1x, K71.3-9, K72, K75, K75.3, K75.9, K76.1, K76.8x, K76.9, K77 |
| Viral hepatitis | B15.x - B19.xx |
| **Chronic liver disease-related Complications** | |
| Acute kidney injury | N17.9, N28.9 |
| Ascites | K70.11, K70.31, K71.51, R18.x |
| Cellulitis | K12.2, L03.01x, L03.03x, L03.11x, L03.211, L03.213, L03.221, L03.31x, L03.81x, L03.90 |
| Cholangitis | K83.0x |
| Encephalopathy | G93.4x, K72.xx |
| Esophageal varices | I85.xx |
| Gastrointestinal hemorrhage | K92.0-2 |
| Hepatorenal syndrome | K76.7 |
| Hydrothorax | J90, J94.8 |
| Hyponatremia | E87.1 |
| Infection (including sepsis, pneumonia) | A04.xx, A08.xx, A09, A40.x, A41.x, A48.1, A48.3, A48.8, A49.0x, A49.1, A49.8-9, B25.0, B44.0, B44.1, B49, B95.xx, B96.xx, J10.0x, J11.0x, J12.xx, J13, J14, J15.xx, J16.x, J17, J18.0-1, J18.9 |
| Jaundice | R17 |
| Liver failure | K70.4x, K72.xx |
| Peritonitis | K65.x, K67, K68.12, K68.19, K68.9 |
| Portal hypertension | K76.6 |
| Portal vein thrombosis or deep vein thrombosis | I80.1x - I80.9, I81, I82.0 - I82.70x, I82.72x, I82.Axx - I82.Cxx, I82.89x, I82.9x |
| Renal failure | N18.x, N19, R94.4 |
| Sepsis | A40.x, A41.x, R65.2x |
| Volume overload or dehydration | E86.0, E87.7x, R60.x |

| Supplementary Table 3. Weighted Prevalence of Sarcopenia and Life's 7 Metrics in Adults with CLD, by the Presence of Sarcopenia, NHANES III (1988-1994), Stratified by Different Liver Diseases | | | | | | | | | | | | | | | |
| --- | --- | --- | --- | --- | --- | --- | --- | --- | --- | --- | --- | --- | --- | --- | --- |
|  | NAFLD | | | HCV | | | HBV | | | ALD | | | Control | | |
|  | Sarcopenia | No Sarcopenia | **p** | Sarcopenia | No Sarcopenia | p | Sarcopenia | No Sarcopenia | p | Sarcopenia | No Sarcopenia | p | Sarcopenia | No Sarcopenia | p |
| **Age, mean (SE)** | 51.94 (0.51) | 41.94 (0.58) | 0 | 45.52 (2.49) | 37.75 (0.76) | 0.0177 | 50.86 (.) | 39.16 (2.28) | 0.0339 | 51.69 (0.95) | 41.03 (1.46) | 0 | 51.16 (0.67) | 39.38 (0.38) | 0 |
| **Males, %** | 49.75 (2.10) | 51.46 (1.81) | 0.6003 | 52.39 (13.54) | 73.73 (3.80) | 0.1062 | 45.67 (9.39) | 69.35 (6.64) | 0.057 | 66.36 (5.51) | 63.90 (3.85) | 0.7016 | 42.81 (1.42) | 49.40 (0.81) | <.0001 |
| **Race, %** |  |  |  |  |  |  |  |  |  |  |  |  |  |  |  |
| non-Hispanic White | 76.58 (1.57) | 74.38 (2.32) | 0.3264 | 63.10 (10.45) | 53.99 (6.51) | 0.3112 | 24.15 (4.32) | 53.14 (7.96) | <.0001 | 80.37 (3.64) | 81.39 (3.31) | 0.825 | 77.18 (1.75) | 77.63 (1.38) | 0.7811 |
| non-Hispanic Black | 10.10 (1.03) | 8.27 (0.76) | 0.0899 | 14.05 (3.77) | 23.88 (3.66) | 0.0316 | 52.15 (9.05) | 20.19 (4.14) | 0.002 | 9.97 (2.62) | 7.25 (1.45) | 0.2678 | 15.23 (1.34) | 9.37 (0.63) | <.0001 |
| Mexican American | 6.07 (0.50) | 6.96 (1.00) | 0.2564 | 5.91 (1.96) | 6.40 (1.65) | 0.8485 | 4.32 (0.77) | 1.32 (0.85) | 0.0509 | 8.08 (1.61) | 5.43 (0.93) | 0.0973 | 3.84 (0.50) | 4.75 (0.42) | 0.0586 |
| Other Race | 7.25 (1.30) | 10.39 (1.55) | 0.0737 | 16.94 (10.49) | 15.74 (6.06) | 0.8636 | 19.38 (11.62) | 25.35 (4.55) | 0.608 | 1.58 (1.14) | 5.93 (2.46) | 0.0547 | 3.75 (0.85) | 8.26 (0.95) | 0.0002 |
| **Income low, %** | 17.61 (1.38) | 17.72 (1.98) | 0.9553 | 42.02 (9.30) | 41.70 (6.73) | 0.9775 | 15.82 (7.38) | 35.96 (8.70) | 0.1381 | 23.28 (5.26) | 15.09 (3.22) | 0.1249 | 17.52 (1.57) | 16.48 (1.19) | 0.4797 |
| **College, %** | 32.65 (1.70) | 40.54 (2.32) | <.0001 | 11.60 (6.75) | 21.94 (6.50) | 0.3068 | 10.97 (1.63) | 45.18 (8.79) | <.0001 | 29.16 (5.07) | 46.21 (4.44) | 0.0086 | 36.29 (1.83) | 47.54 (1.52) | <.0001 |
| **Married, %** | 70.47 (2.08) | 72.41 (1.55) | 0.353 | 61.27 (6.76) | 45.02 (4.97) | 0.0412 | 70.71 (8.11) | 59.04 (11.21) | 0.4448 | 55.42 (5.67) | 66.60 (5.72) | 0.1363 | 67.56 (1.62) | 67.50 (1.05) | 0.976 |
| **Sarcopenia, %** | 100.00 (0.00) | 0 |  | 100.00 (0.00) | 0 |  | 100.00 (0.00) | 0 |  | 100.00 (0.00) | 0 |  | 100.00 (0.00) | 0 |  |
| **Life Simple (LS) Metric** |  |  |  |  |  |  |  |  |  |  |  |  |  |  |  |
| **Total Serum Cholesterol, mg/dL** |  |  |  |  |  |  |  |  |  |  |  |  |  |  |  |
| Ideal (<200 untreated) | 30.39 (1.92) | 51.25 (2.09) | <.0001 | 73.58 (8.24) | 70.31 (4.43) | 0.7153 | 69.31 (5.70) | 59.29 (9.30) | <.0001 | 31.72 (4.84) | 40.90 (4.23) | 0.181 | 31.40 (1.25) | 57.64 (1.14) | <.0001 |
| Intermediate (200-239 or treated) | 41.81 (1.85) | 30.88 (1.62) | <.0001 | 20.07 (3.61) | 24.92 (4.30) | 0.4326 | 10.23 (1.83) | 33.77 (10.28) | <.0001 | 38.49 (5.34) | 32.11 (4.68) | 0.383 | 39.09 (1.64) | 28.33 (0.89) | <.0001 |
| Poor (≥ 240) | 27.80 (1.44) | 17.86 (1.45) | <.0001 | 6.35 (6.13) | 4.76 (1.98) | 0.7858 | 20.46 (4.13) | 6.94 (4.40) | 0.1137 | 29.79 (4.35) | 26.99 (4.12) | 0.6551 | 29.51 (1.49) | 14.02 (0.77) | <.0001 |
| **Glycemic control, HbA1c *** |  |  |  |  |  |  |  |  |  |  |  |  |  |  |  |
| Ideal (< 5.7%) | 58.45 (2.30) | 79.79 (1.47) | <.0001 | 68.55 (10.48) | 75.68 (5.49) | 0.4568 | 62.06 (7.10) | 91.32 (3.28) | 0.0001 | 75.89 (5.00) | 92.01 (2.59) | 0.0026 | 74.74 (1.68) | 88.61 (0.91) | <.0001 |
| Intermediate (5.7 - 6.4) | 26.99 (1.95) | 13.77 (1.03) | <.0001 | 9.46 (6.53) | 21.57 (5.68) | 0.1043 | 35.84 (6.80) | 5.92 (3.07) | 0.0002 | 13.73 (3.72) | 6.87 (2.46) | 0.0947 | 20.89 (1.52) | 9.57 (0.83) | <.0001 |
| Poor (≥ 6.5%) | 14.55 (1.33) | 6.43 (1.05) | <.0001 | 21.99 (8.18) | 2.75 (0.55) | <.0001 | 2.09 (0.37) | 2.76 (0.48) | 0.4119 | 10.39 (3.28) | 1.12 (0.60) | <.0001 | 4.37 (0.43) | 1.82 (0.28) | <.0001 |
| **Smoking Status** |  |  |  |  |  |  |  |  |  |  |  |  |  |  |  |
| Ideal | 40.60 (1.98) | 44.46 (1.75) | 0.1253 | 42.99 (14.61) | 11.65 (3.54) | 0.0007 | 52.32 (9.14) | 55.76 (8.99) | 0.8331 | 20.89 (4.31) | 26.60 (4.46) | 0.2565 | 40.67 (2.13) | 43.75 (1.30) | 0.2178 |
| Intermediate | 41.72 (2.07) | 27.07 (1.66) | <.0001 | 12.24 (9.88) | 19.61 (7.03) | 0.5657 | 23.30 (4.45) | 17.90 (11.10) | 0.6262 | 41.83 (6.18) | 26.99 (4.97) | 0.0374 | 31.19 (1.58) | 22.08 (0.80) | <.0001 |
| Poor | 17.68 (1.41) | 28.47 (1.62) | <.0001 | 44.77 (11.61) | 68.74 (6.46) | 0.0337 | 24.38 (11.65) | 26.34 (5.49) | 0.8436 | 37.29 (6.16) | 46.41 (4.29) | 0.2316 | 28.14 (1.71) | 34.17 (1.15) | 0.0047 |
| **Blood Pressure, mm Hg** |  |  |  |  |  |  |  |  |  |  |  |  |  |  |  |
| Ideal (<120/80 untreated) | 19.31 (1.72) | 48.94 (1.75) | <.0001 | 25.77 (5.66) | 48.89 (7.45) | 0.0302 | 32.57 (5.29) | 70.46 (6.11) | <.0001 | 9.53 (2.54) | 41.92 (5.52) | <.0001 | 27.51 (1.69) | 58.24 (1.12) | <.0001 |
| Intermediate (120-129/80 or treated) | 18.10 (1.44) | 13.81 (1.02) | 0.018 | 19.86 (7.97) | 12.14 (3.20) | 0.3777 | 8.47 (1.51) | 8.15 (1.50) | 0.897 | 20.78 (5.59) | 12.58 (2.61) | 0.0974 | 17.63 (1.36) | 13.82 (0.74) | 0.0059 |
| Poor (≥ 130/80) | 62.59 (2.18) | 37.24 (1.84) | <.0001 | 54.37 (7.65) | 38.97 (5.86) | 0.1657 | 58.96 (5.29) | 21.39 (5.45) | <.0001 | 69.69 (5.90) | 45.50 (5.06) | 0.0041 | 54.87 (1.91) | 27.94 (1.03) | <.0001 |
| **Healthy Diet Score**** |  |  |  |  |  |  |  |  |  |  |  |  |  |  |  |
| Ideal (>69.3) | 36.25 (2.27) | 34.29 (1.88) | 0.5135 | 17.09 (10.39) | 28.90 (7.79) | 0.3034 | 47.14 (5.73) | 21.38 (5.66) | 0.0002 | 40.56 (6.21) | 21.34 (3.90) | 0.005 | 30.45 (1.67) | 33.30 (1.30) | 0.1314 |
| Intermediate (56.9-69.3) | 32.45 (1.65) | 33.56 (1.38) | 0.6165 | 46.96 (12.58) | 29.84 (4.75) | 0.1332 | 35.94 (7.81) | 53.58 (11.03) | 0.0126 | 23.66 (4.01) | 39.30 (4.88) | 0.0269 | 35.05 (2.40) | 35.10 (1.05) | 0.9838 |
| Poor (<56.9) | 31.30 (2.21) | 32.15 (1.62) | 0.7583 | 35.95 (11.37) | 41.26 (5.65) | 0.6617 | 16.92 (5.27) | 25.04 (5.55) | 0.1228 | 35.78 (4.91) | 39.36 (4.99) | 0.6143 | 34.50 (1.90) | 31.60 (1.05) | 0.1704 |
| **Body Mass Index** |  |  |  |  |  |  |  |  |  |  |  |  |  |  |  |
| Ideal (BMI <25) | 2.52 (0.48) | 44.31 (1.91) | <.0001 | 1.91 (1.48) | 70.89 (5.01) | <.0001 | 8.88 (7.68) | 77.60 (5.69) | <.0001 | 2.71 (1.43) | 43.17 (4.06) | <.0001 | 12.04 (1.37) | 61.87 (1.11) | <.0001 |
| Intermediate (BMI 25-29) | 25.51 (1.95) | 38.59 (1.78) | <.0001 | 26.90 (12.26) | 19.56 (4.58) | 0.6055 | 37.67 (8.39) | 21.86 (5.67) | 0.1031 | 33.07 (5.47) | 42.95 (4.32) | 0.1429 | 37.36 (1.78) | 31.76 (0.82) | 0.0068 |
| Poor (BMI ≥ 30) | 71.97 (2.12) | 17.09 (1.67) | <.0001 | 71.18 (12.48) | 9.55 (1.95) | <.0001 | 53.45 (6.17) | 0.54 (0.09) | <.0001 | 64.23 (5.36) | 13.88 (2.63) | <.0001 | 50.60 (2.22) | 6.37 (0.53) | <.0001 |
| **Physical Activity** |  |  |  |  |  |  |  |  |  |  |  |  |  |  |  |
| Ideal | 30.73 (2.25) | 42.11 (2.15) | <.0001 | 38.42 (7.78) | 40.83 (5.51) | 0.7992 | 44.20 (6.18) | 51.18 (6.79) | 0.4691 | 33.01 (5.63) | 40.63 (4.46) | 0.3015 | 35.07 (2.43) | 43.90 (1.37) | 0.0002 |
| Intermediate | 51.49 (1.90) | 44.20 (2.31) | 0.0016 | 45.16 (7.12) | 39.87 (6.41) | 0.5443 | 32.68 (6.57) | 25.95 (7.02) | 0.512 | 47.00 (5.78) | 47.42 (3.95) | 0.9502 | 46.90 (2.00) | 45.68 (1.10) | 0.5738 |
| Poor | 17.78 (1.35) | 13.69 (1.28) | 0.005 | 16.42 (3.95) | 19.30 (4.56) | 0.665 | 23.12 (4.84) | 22.87 (5.16) | 0.9595 | 20.00 (4.45) | 11.95 (2.93) | 0.0848 | 18.02 (1.72) | 10.42 (0.79) | <.0001 |
| **N. of Ideal LS Metrics** |  |  |  |  |  |  |  |  |  |  |  |  |  |  |  |
| 0 | 8.05 (0.92) | 2.74 (0.56) | <.0001 | 0.73 (0.73) | 0 |  | 0 | 0 |  | 7.23 (2.31) | 1.16 (0.70) | 0.0029 | 3.91 (0.60) | 1.07 (0.20) | <.0001 |
| 1 | 21.73 (1.48) | 9.33 (1.34) | <.0001 | 10.68 (2.65) | 5.79 (1.41) | 0.0565 | 23.28 (4.54) | 0 | <.0001 | 22.70 (4.71) | 11.49 (3.17) | 0.0099 | 17.66 (1.40) | 4.32 (0.49) | <.0001 |
| 2 | 31.16 (1.38) | 17.32 (1.47) | <.0001 | 22.94 (8.07) | 15.26 (3.23) | 0.2791 | 15.40 (4.48) | 6.85 (2.01) | 0.1098 | 35.03 (5.89) | 24.05 (5.88) | 0.2045 | 29.28 (1.71) | 12.42 (0.62) | <.0001 |
| 3 | 25.00 (1.79) | 20.81 (1.43) | 0.0864 | 49.69 (9.54) | 29.26 (3.71) | 0.0544 | 17.82 (3.78) | 14.30 (5.37) | 0.552 | 20.67 (5.18) | 24.22 (4.05) | 0.5963 | 27.31 (1.66) | 21.31 (0.99) | 0.0044 |
| 4 | 11.61 (1.18) | 22.92 (1.78) | <.0001 | 15.97 (8.37) | 29.51 (6.11) | 0.2137 | 16.79 (7.44) | 44.84 (11.72) | 0.0028 | 11.39 (3.88) | 22.69 (3.64) | 0.04 | 15.60 (1.29) | 26.67 (0.79) | <.0001 |
| 5 | 2.29 (0.54) | 17.06 (1.42) | <.0001 | 0 | 13.17 (4.50) |  | 18.46 (7.52) | 20.37 (6.27) | 0.8391 | 2.08 (1.48) | 13.09 (3.06) | 0.0025 | 5.50 (0.92) | 20.49 (0.87) | <.0001 |
| 6 | 0.16 (0.12) | 6.89 (0.79) | <.0001 | 0 | 6.55 (2.19) |  | 8.24 (1.47) | 7.27 (4.37) | 0.8593 | 0.89 (0.89) | 3.31 (1.69) | 0.1768 | 0.76 (0.31) | 10.96 (0.81) | <.0001 |
| 7 | 0 | 2.93 (0.54) |  | 0 | 0.46 (0.32) |  | 0 | 6.37 (5.57) | <.0001 | 0 | 0 |  | 0 | 2.77 (0.42) |  |
| **Elevated AST** | 8.46 (0.82) | 6.96 (0.96) | 0.2352 | 62.82 (10.84) | 48.94 (5.96) | 0.1602 | 18.39 (5.23) | 27.70 (11.07) | 0.0352 | 22.82 (5.01) | 22.74 (3.36) | 0.9882 | 3.26 (0.59) | 2.38 (0.32) | 0.0857 |
| **Elevated ALT** | 11.09 (1.26) | 9.05 (1.17) | 0.2095 | 58.42 (13.25) | 42.02 (6.66) | 0.1597 | 15.86 (4.77) | 25.82 (11.33) | 0.0385 | 30.30 (5.22) | 17.96 (4.27) | 0.0489 | 3.16 (0.79) | 2.89 (0.43) | 0.7532 |
| **Elevated liver enzyme** | 13.40 (1.18) | 10.61 (1.01) | 0.0578 | 68.61 (12.80) | 55.82 (5.05) | 0.3389 | 18.39 (5.23) | 27.70 (11.07) | 0.0352 | 38.92 (5.70) | 31.40 (4.51) | 0.2633 | 4.46 (0.82) | 3.90 (0.41) | 0.4768 |
| **FIB-4, %** |  |  |  |  |  |  |  |  |  |  |  |  |  |  |  |
| FIB4_HIGH | 1.51 (0.31) | 0.80 (0.19) | 0.0273 | 20.50 (8.04) | 7.16 (1.95) | 0.0381 | 0 | 9.06 (4.71) | <.0001 | 8.89 (4.17) | 4.91 (1.51) | 0.1818 | 1.26 (0.25) | 0.91 (0.16) | 0.1934 |
| FIB4_MIDDLE | 22.55 (1.63) | 13.90 (1.02) | <.0001 | 23.60 (6.40) | 14.04 (2.89) | 0.1016 | 21.74 (6.47) | 26.53 (11.03) | 0.762 | 26.53 (5.32) | 17.77 (4.12) | 0.0941 | 23.49 (1.42) | 12.03 (0.72) | <.0001 |
| FIB4_LOW | 75.94 (1.61) | 85.30 (1.05) | <.0001 | 55.90 (8.19) | 78.81 (3.87) | 0.0004 | 78.26 (6.47) | 64.40 (10.58) | 0.3103 | 64.59 (6.05) | 77.32 (4.16) | 0.0477 | 75.25 (1.49) | 87.07 (0.77) | <.0001 |
| **CKD** | 16.65 (1.38) | 8.38 (0.66) | <.0001 | 19.05 (9.92) | 6.55 (1.84) | 0.0911 | 21.15 (6.68) | 0.58 (0.11) | <.0001 | 12.60 (3.16) | 8.05 (2.33) | 0.1022 | 11.79 (1.09) | 6.62 (0.29) | <.0001 |
| **High Risk for CVD** | 42.62 (1.56) | 23.45 (1.34) | <.0001 | 28.57 (9.04) | 9.69 (2.91) | 0.0146 | 41.48 (5.60) | 9.51 (1.66) | <.0001 | 50.28 (5.47) | 20.41 (3.90) | <.0001 | 37.62 (1.66) | 14.65 (0.83) | <.0001 |
| **History of, %** |  |  |  |  |  |  |  |  |  |  |  |  |  |  |  |
| Cancer | 8.52 (0.89) | 5.24 (0.67) | 0.0054 | 11.97 (9.42) | 5.63 (1.39) | 0.3166 | 2.12 (2.11) | 0.99 (0.17) | 0.4144 | 13.39 (4.19) | 4.04 (1.32) | 0.0005 | 9.51 (1.10) | 5.34 (0.43) | <.0001 |
| CVD | 9.93 (0.89) | 3.66 (0.55) | <.0001 | 15.32 (8.19) | 1.68 (0.87) | 0.0013 | 0 | 0.48 (0.50) |  | 6.50 (2.56) | 3.29 (1.80) | 0.2804 | 6.85 (0.89) | 2.39 (0.31) | <.0001 |
| Family CVD | 18.24 (1.24) | 18.11 (0.97) | 0.9224 | 26.88 (10.08) | 21.27 (3.30) | 0.5516 | 22.66 (4.39) | 9.69 (1.78) | 0.0022 | 21.82 (5.57) | 17.36 (2.92) | 0.3923 | 16.03 (1.89) | 16.91 (0.76) | 0.6919 |
| **Cumulative Mortality, %** |  |  |  |  |  |  |  |  |  |  |  |  |  |  |  |
| All causes | 42.35 (1.54) | 20.76 (1.38) | <.0001 | 57.09 (14.73) | 28.86 (4.61) | 0.0544 | 32.20 (6.67) | 20.69 (7.52) | 0.2392 | 53.62 (5.14) | 27.67 (3.28) | <.0001 | 37.15 (2.05) | 17.01 (0.94) | <.0001 |
| CVD | 10.51 (0.94) | 5.15 (0.63) | <.0001 | 0.59 (0.61) | 7.20 (2.44) | 0.0025 | 0 | 0 |  | 10.75 (4.36) | 6.58 (2.22) | 0.2618 | 9.89 (0.84) | 4.20 (0.34) | <.0001 |
| Cancer | 9.14 (0.85) | 5.60 (0.88) | 0.0011 | 5.56 (1.94) | 3.91 (2.48) | 0.6262 | 14.10 (3.74) | 0.68 (0.12) | <.0001 | 22.11 (4.95) | 9.40 (2.82) | 0.0082 | 8.73 (1.09) | 4.94 (0.39) | 0.0001 |
| Diabetes | 2.65 (0.56) | 0.47 (0.17) | <.0001 | 2.49 (1.65) | 2.23 (0.74) | 0.8764 | 0 | 4.72 (4.49) |  | 0.38 (0.15) | 0.57 (0.41) | 0.6339 | 0.54 (0.19) | 0.23 (0.08) | 0.0279 |
| Abbreviation: CVD, Cardiovascular disease; SE, standard error. * Hemoglobin A1c values were used as proxies for blood glucose metrics. ** Healthy diet score was calculated based on the 2010-healthy eating index recommended by Dietary Guidelines for Americans.  All values were displayed as weighted percentages (95% confidence interval) except where otherwise noted. Mortality status followed up to December 2015. | | | | | | | | | | | | | | | |
